# Extrachromosomal circular DNA drives focal amplification of AGAP2-AS1 to promote cutaneous tumorigenesis

**DOI:** 10.3389/fphar.2026.1906797

**Published:** 2026-10-14

**Authors:** Jin Zhang, Zeying Liu, Jinzhi Zhao, Xun Liu, Zhiying Liu, Huiting Xue, Tingting Lin, Limin Zhu

**Affiliations:** 1 Tianjin Key Laboratory of Retinal Functions and Diseases, Tianjin Branch of National Clinical Research Center for Ocular Disease, Eye Institute and School of Optometry, Tianjin Medical University Eye Hospital, Tianjin, China; 2 Department of Ophthalmology, The First Hospital of Hohhot, Hohhot, China; 3 Tumor Multimodal Digital and Intelligent Precision Diagnosis Engineering Research Center, School of Basic Medical Sciences, Inner Mongolia Medical University, Hohhot, China

**Keywords:** AGAP2-AS1, cutaneous tumorigenesis, extrachromosomal circular DNA, eyelid neoplasm, gene amplification

## Abstract

**Background:**

Extrachromosomal circular DNA (eccDNA) represents a pervasive structural continuum of genomic instability that drives intratumoral heterogeneity; the topography of the eccDNA-encoded non-coding transcriptome remains obscure in ultraviolet (UV)-driven malignancies.

**Methods:**

We profiled eccDNA from Basal Cell Carcinoma, Inverted Follicular Keratosis, Merkel Cell Carcinoma, and matched adjacent normal tissues using Circle-Seq. Structural data were integrated with the eccDNA Gene Analyzer (ECGA) and The Cancer Genome Atlas (TCGA) clinical cohorts. In vitro siRNA-mediated knockdown assays were used to determine the oncogenic functionality of identified lncRNAs.

**Results:**

Circle-Seq revealed genome-wide remodeling of circular DNA with focal hyper-amplification, GC-rich compositional biases, and recurrent hotspots on chromosomes 9, 16, and 20. Integrative screening identified AGAP2-AS1 as a clinically significant, copy-number-associated lncRNA, whose expression correlated with cellular responses to UV radiation and oxidative stress. Depletion of AGAP2-AS1 impaired tumor cell proliferation, migration, and invasion in vitro.

**Conclusion:**

The eccDNA landscape serves as a reservoir of functional non-coding elements; focal amplification of AGAP2-AS1 contributes to malignant progression and stress adaptation in cutaneous neoplasia.

## 1 Introduction

Genomic plasticity serves as a fundamental hallmark of neoplastic evolution, enabling tumors to navigate environmental pressures and therapeutic insults (Hanahan, 2022). While chromosomal structural variations (SVs) have historically dominated the characterization of cancer genomes, extrachromosomal circular DNA (eccDNA) represents a distinct, pervasive reservoir of focal hyper-amplification. In this study, we conceptualize eccDNA as a continuous structural spectrum, encompassing elements ranging from small, sub-genic “microDNAs” (<400 bp) to megabase-sized, autonomously replicating “ecDNAs” (extrachromosomal DNAs) capable of harboring full-length oncogenes and regulatory units (Luebeck et al., 2023; Shibata et al., 2012). Unlike linear chromosomal loci, these circular topologies lack centromeres and segregate unequally during mitosis, driving rapid intercellular heterogeneity and high-copy-number amplification independent of the host genome (Wu et al., 2019). Despite their potent role in accelerating tumor evolution, the comprehensive topography of the eccDNA landscape—particularly the non-coding transcriptome carried by these mobile elements—remains underexplored in cutaneous malignancies.

Periocular cutaneous tumors constitute a unique genomic archetype defined by chronic ultraviolet (UV) exposure. Arising in a microenvironment subject to continuous genotoxic stress, these malignancies harbor high burdens of UV-signature mutations and double-strand breaks (DSBs), which are mechanistic prerequisites for DNA circularization and “chromothripsis” events (Alexandrov et al., 2013). Although focal amplifications of driver genes are well-documented in skin cancers, the contribution of extrachromosomal circularization to these copy number alterations (CNVs) is poorly defined. Specifically, it remains obscure whether the eccDNA repertoire in this high-stress context is shaped by stochastic genomic shattering or by the selective retention of functional regulatory units—such as long non-coding RNAs (lncRNAs)—that modulate adaptive stress responses (Huarte, 2015).

Circle-Seq exploits plasmid-safe ATP-dependent DNase to selectively digest linear chromosomal DNA while preserving circular topologies, thereby enabling high-specificity enrichment of the eccDNA fraction with substantially reduced linear background contamination compared to conventional whole-genome sequencing approaches (Møller, 2020). Beyond simple oncogene dosage amplification, eccDNAs can mobilize distal regulatory elements and lncRNAs that function as molecular decoys or transcriptional modulators, rewiring oncogenic signaling networks through both cis- and trans-acting mechanisms (Helmsauer et al., 2020; Paulsen et al., 2019). Notably, the lncRNA AGAP2-AS1 is an established oncogenic driver in glioblastoma and non-small cell lung cancer (NSCLC) (Li et al., 2016; Schmitt and Chang, 2016), yet whether such drivers can be structurally co-opted through eccDNA-mediated focal amplification in the context of chronic UV-induced genomic instability has not been investigated.

Identifying these non-coding drivers requires an integrative approach that couples structural identification with clinical outcome data. Here, we define the landscape of genomic instability in periocular malignancies by integrating in-house Circle-Seq profiling with large-scale genomic data. To capture the broad spectrum of eyelid tumorigenesis, our discovery cohort encompasses diverse pathological subtypes, including Basal Cell Carcinoma (BCC), Inverted Follicular Keratosis (IFK), and Merkel Cell Carcinoma (MCC). By cross-referencing this unique dataset with the specialized eccDNA Gene Analyzer (ECGA) database and validating clinical relevance in The Cancer Genome Atlas (TCGA) Skin Cutaneous Melanoma (SKCM) cohort, we establish a multi-source screening framework. We identify a specific repertoire of copy-number-associated, tumor-enriched lncRNAs, providing evidence that eccDNA formation is not merely a byproduct of genomic instability but likely serves as an adaptive mechanism that facilitates malignant progression.

## 2 Results

### 2.1 Global remodeling of the extrachromosomal circular DNA landscape in eyelid cutaneous tumors

To define the topography of genomic instability mediated by extrachromosomal circular DNA (eccDNA) in periocular malignancies, we profiled paired eyelid cutaneous tumor tissues and matched adjacent non-neoplastic tissues using Circle-Seq. Specifically, our cohort consisted of three distinct pathological subtypes: Basal Cell Carcinoma (BCC, n = 1), Inverted Follicular Keratosis (IFK, n = 1), and Merkel Cell Carcinoma (MCC, n = 1), along with their corresponding adjacent controls. This paired tumor-normal design enables intra-individual normalization, effectively controlling for background genetic variation, age-related somatic eccDNA accumulation, and environmental confounders that would otherwise obscure tumor-specific signals. While the limited sample size constrains subtype-level statistical inference, the pan-histological scope of the cohort was intentionally designed to capture shared eccDNA features across biologically distinct cutaneous tumor entities arising within a common UV-exposed periocular microenvironment. Eyelid tumors harbored a marked, albeit heterogeneous, expansion of eccDNA burden relative to adjacent controls (Figure 1A), with the BCC sample exhibiting the most substantial increase in eccDNA count. Fragment size distributions in both cohorts displayed characteristic periodicity peaking at ∼180 bp and ∼360 bp, consistent with nucleosomal protection and confirming the specific recovery of circular species (Figure 1B). Beyond global abundance, tumor-derived eccDNAs exhibited a higher median GC content compared to adjacent tissues, although this trend did not reach statistical significance in the rigorous sample-level analysis (Figure 1C). While eccDNAs mapped broadly across distinct functional elements—predominantly originating from intronic and distal intergenic regions in both groups (Figure 1D)—principal component analysis (PCA) of genome-wide abundance revealed a segregation of neoplastic profiles from their non-tumor counterparts (PC1: 93.6% variance). Notably, the variance captured by PC1 was primarily driven by the BCC sample, highlighting the substantial inter-tumoral heterogeneity within the eccDNA landscape alongside the systemic reshaping associated with eyelid tumorigenesis (Figure 1E).

**FIGURE 1:**
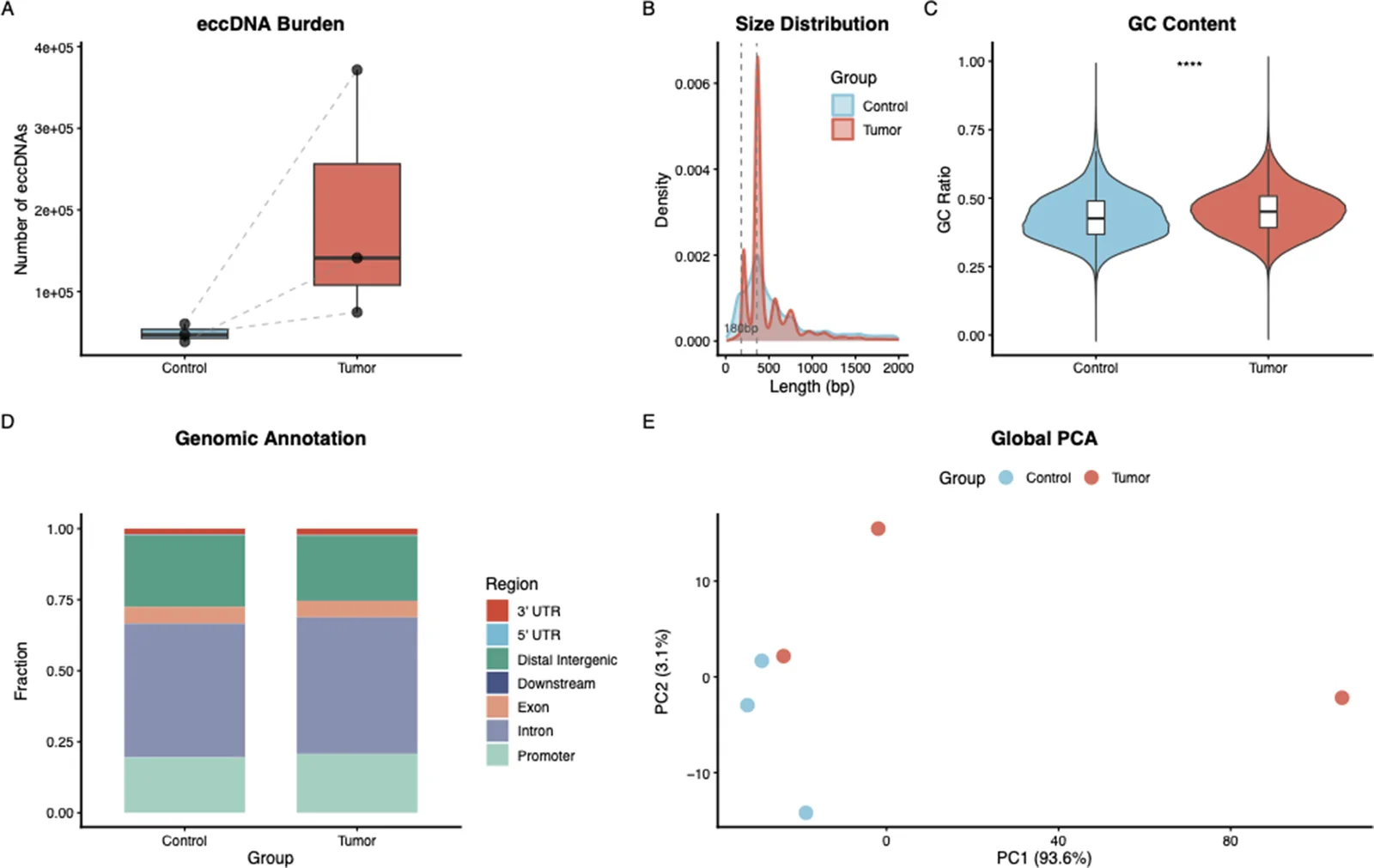
Global characterization and genomic landscape of eccDNAs in eyelid cutaneous tumors versus adjacent tissues. **(A)** Box plots showing the global burden of unique eccDNAs identified in eyelid cutaneous tumor tissues (n = 3) and matched adjacent normal tissues (n = 3). Individual biologically independent samples are labeled according to their pathological subtype (BCC, IFK, MCC); dashed lines connect paired samples. The center line denotes the median, box limits indicate the upper and lower quartiles, and whiskers extend to 1.5× interquartile range. **(B)** Density profiles displaying the length distribution of eccDNAs. The vertical dashed line at ∼180 bp indicates the length of DNA wrapped around a single nucleosome, confirming the purification of circular DNA species. **(C)** Box and jitter plots quantifying the sample-level average GC content ratio of eccDNAs in adjacent control (blue) and tumor (red) groups. Individual data points (N = 3 per group) are displayed and labeled. Statistical significance was determined by a two-tailed Student’s t-test (ns = not significant). **(D)** Stacked bar charts illustrating the genomic distribution of eccDNAs across distinct functional elements. **(E)** Principal component analysis (PCA) of genome-wide eccDNA profiles. The plot shows separation between eyelid tumors (red) and adjacent normal tissues (blue), with PC1 capturing 93.6% of the total variance, predominantly driven by the BCC sample.

### 2.2 Spatial topology and focal hyper-amplification of the eccDNA landscape

Mapping the genomic topography of eccDNA biogenesis revealed a pervasive yet spatially heterogeneous amplification landscape in eyelid cutaneous tumors. Circos visualization highlights distinct high-density eccDNA clusters in tumor genomes that contrast sharply with the relatively uniform distribution observed in matched adjacent non-neoplastic tissues (Figure 2A). This pan-chromosomal elevation is substantiated by length-normalized density analysis, which demonstrates that while eccDNA burden is elevated across almost all autosomes, specific chromosomes exhibit disproportionately high generation rates relative to their genomic size (Figure 2B). Analysis of inter-eccDNA distances uncovers distinct genomic instability features reminiscent of kataegis (Bergstrom et al., 2022). To definitively separate the signals, we visualized the inter-eccDNA distances of the tumor and control groups in parallel tracks. Tumor genomes displayed localized, high-frequency eccDNA clusters (inter-eccDNA distance <10^4 bp) that were substantially enriched relative to the sparser background distribution observed in the control group, indicative of coordinated genomic shattering events (Figure 2C). Further resolution of differential abundance at 1-Mb windows identifies specific genomic hotspots driving this instability. Subtraction of the background signal reveals dominant, tumor-specific amplification peaks converging on loci within chromosomes 9, 16, and 20, suggesting that eccDNA formation in this context is driven by locus-specific selection pressures rather than stochastic genomic degradation (Figure 2D).

**FIGURE 2:**
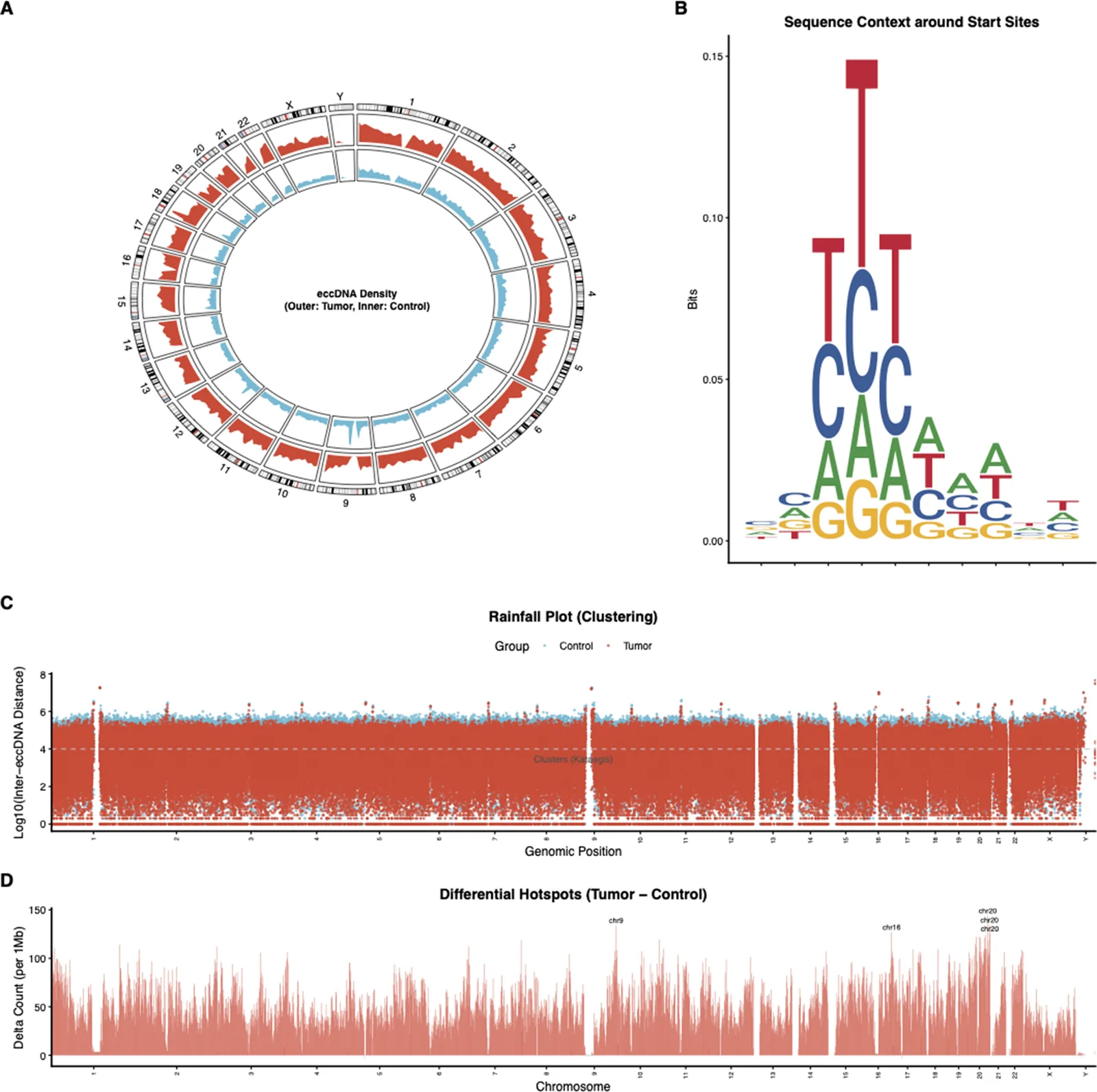
Chromosomal topography and focal genomic instability of eccDNAs in eyelid cutaneous tumors. **(A)** Circos plot illustrating the genome-wide density of eccDNAs. The outer track (red) represents eyelid tumor samples, showing distinct high-density regions compared to the inner track (blue) representing matched adjacent normal tissues. **(B)** Bar chart quantifying the chromosomal distribution of eccDNAs normalized by chromosome length (count per 100 Mb). Data are stratified by group (Tumor: red; Control: blue). **(C)** Rainfall plot depicting genomic instability and clustering events. The y-axis represents the log10 inter-eccDNA distance; the x-axis represents the genomic position. Data points for Tumor (red) and Control (blue) groups are displayed in separate facet panels to prevent visual overlap. Dots falling below the dashed threshold (10^4 bp) indicate kataegis-like focal hyper-amplification clusters, which are markedly enriched in tumors compared to the background distribution in controls. **(D)** Manhattan-style plot displaying the differential eccDNA burden (Tumor minus Control) across the genome in 1-Mb windows. The y-axis represents the delta count, highlighting specific chromosomal hotspots (labeled) on chromosomes 9, 16, and 20 that undergo preferential circularization in tumors.

### 2.3 Recurrent amplification of structural and regulatory elements via eccDNAs

Differential abundance profiling of the eyelid tumor eccDNA landscape reveals a profound skew towards focal amplification. A broad repertoire of 21,649 tumor-enriched eccDNA-associated genes is identified (log_2(FC) > 1, high abundance), contrasting sharply with a limited set of only 134 depleted loci (Figure 3A). Motif analysis of circularization junctions uncovers a distinct sequence signature at breakpoint boundaries, implying that a sequence-dependent enzymatic mechanism—rather than stochastic fragmentation—drives the excision of these tumor-specific circles (Figure 3B).

**FIGURE 3:**
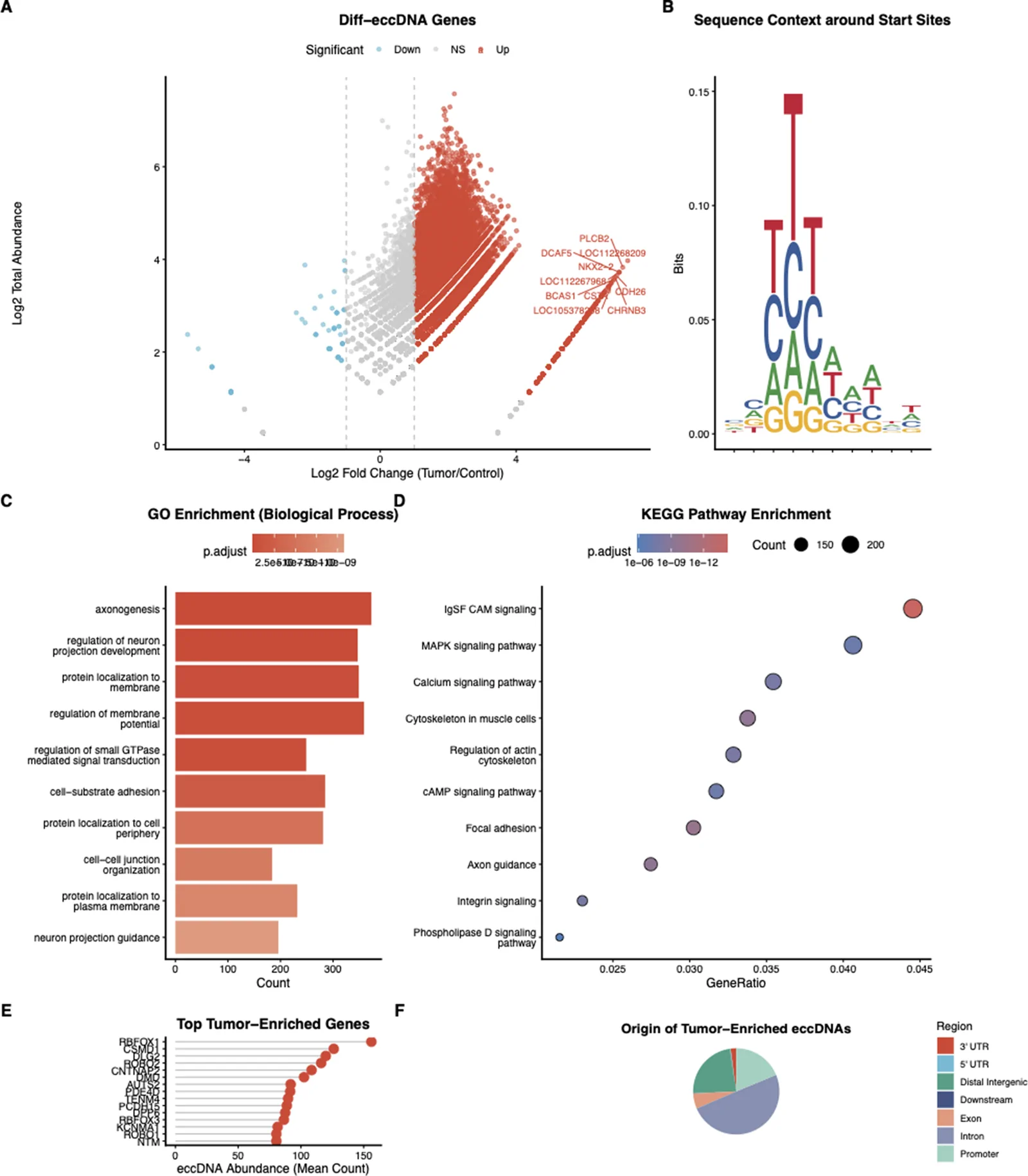
Functional genomic signature and mechanistic origins of tumor-enriched eccDNAs in eyelid cutaneous tumors. **(A)** Volcano plot displaying differential eccDNA abundance between eyelid tumor and matched adjacent normal samples. Red points indicate significantly upregulated (tumor-enriched) loci (n = 21,649), while blue points indicate downregulated loci (n = 134). **(B)** SeqLogo visualization of the sequence context flanking eccDNA junction sites (breakpoints), revealing conserved motif features at the circularization boundaries. **(C)** Bar chart summarizing the top enriched Gene Ontology (GO) biological processes associated with tumor-specific eccDNAs, ranked by adjusted P-value. Full GO enrichment results are provided in Supplementary Table S3. **(D)** Lollipop chart ranking the top 15 genes with the highest eccDNA-mediated absolute amplification burden in tumors (e.g., RBFOX1, CSMD1, ROBO2). Data are presented as mean eccDNA counts. **(E)** Pie chart illustrating the genomic feature distribution of tumor-enriched eccDNAs, showing a predominant bias towards intronic (49.7%) and distal intergenic (23.4%) regions.

Functional interrogation of genes harbored on these amplified eccDNAs unveils a significant enrichment in biological processes governing cellular architecture and environment interactions. Gene Ontology (GO) analysis highlights processes such as protein localization to the membrane, cell-substrate adhesion, and cell-cell junction organization (Figure 3C; full results in Supplementary Table S3). Notably, pathway analysis did not yield significantly enriched canonical metabolic cascades, suggesting that the functional impact of eccDNA amplification in this specific context may be predominantly tied to structural and adhesive modulations rather than broad metabolic network rewiring.

Dominant amplified loci encompass large genomic-spanning genes such as RBFOX1, CSMD1, and ROBO2 (Figure 3D)—loci frequently susceptible to replication stress. Crucially, genomic annotation demonstrates that these tumor-enriched eccDNAs predominantly originate from non-coding regions, specifically intronic (49.7%) and distal intergenic (23.4%) sequences (Figure 3E). This strong non-coding bias supports the hypothesis that they primarily function as mobile enhancer elements or regulatory sponges reshaping the tumor microenvironment, rather than solely as coding amplicons.

### 2.4 Dual-source integrative screening identifies *AGAP2-AS1* as a clinically significant, copy-number-driven oncogene

To systematically identify clinically relevant non-coding drivers originating from extrachromosomal circular DNA (eccDNA), we implemented a multi-tiered screening strategy integrating our in-house Circle-Seq profiling with the eccDNA gene analyzer (ECGA) database (https://www.zhounan.org/ecga/). We first defined a high-confidence repertoire of circle-encoded non-coding RNAs by intersecting our eyelid tumor-derived peaks with the extensive eccDNA catalogue from ECGA.

To determine the clinical significance of this filtered repertoire, given that both eyelid malignancies and melanoma are fundamentally driven by chronic ultraviolet (UV) exposure and share a highly analogous genotoxic microenvironment, we leveraged the TCGA Skin Cutaneous Melanoma (SKCM) cohort as an optimal surrogate for transcriptomic and prognostic interrogation. Univariate Cox proportional hazards regression analysis pinpointed three long non-coding RNAs—*AGAP2-AS1*, LINC01780, and LINC01537—as predictors of adverse progression-free survival (Hazard Ratio >1, P < 0.05; Figure 4A). A risk-stratified heatmap, constructed using risk scores derived from the Cox regression coefficients, confirmed the specific upregulation of these candidates in high-risk subgroups (Figure 4B). To explore their maximal prognostic divergence, stratification thresholds were determined using maximally selected rank statistics (optimal cutoff). The optimal expression cutoffs for the three lncRNAs are illustrated in Figures 4F–H. Elevated expression of *AGAP2-AS1*, LINC01780, and LINC01537 correlated with shortened progression-free survival (Figures 4C–E). It should be noted that while this data-driven approach optimizes cohort separation, it inherently inflates statistical significance, and therefore these Kaplan-Meier estimates should be interpreted as exploratory prognostic trends rather than confirmatory independent predictors.

**FIGURE 4:**
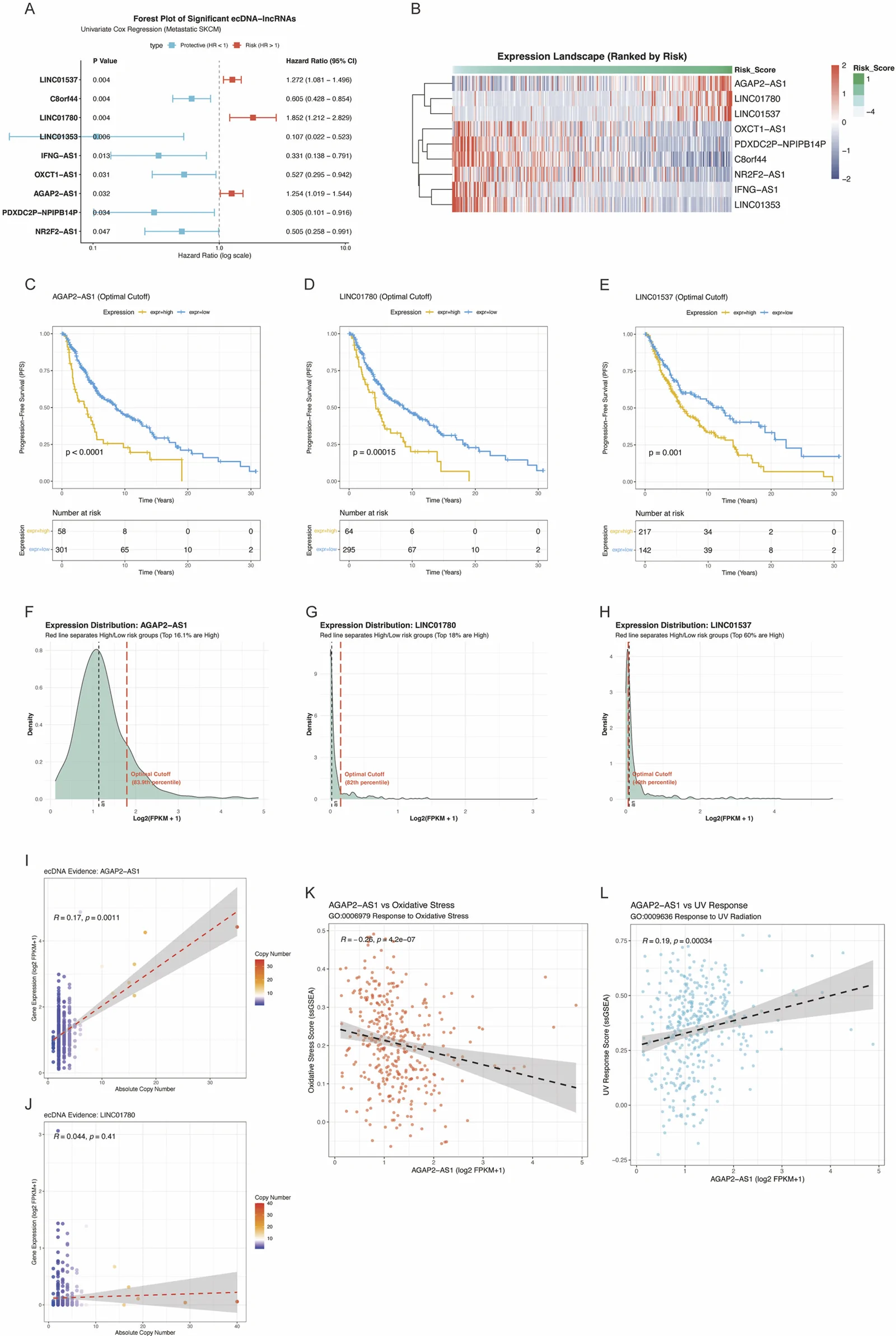
Integrative screening and functional characterization of *AGAP2-AS1* as an eccDNA-driven oncogenic lncRNA. **(A)** Forest plot of univariate Cox regression analysis identifying significant eccDNA-associated lncRNAs. The candidate set was defined by intersecting in-house Circle-Seq profiles (eyelid tumors) with the ECGA database (eccDNA-specific), followed by prognostic validation in the TCGA-SKCM cohort. Hazard ratios (HR) and 95% confidence intervals are shown; red indicates risk factors (HR > 1). **(B)** Heatmap visualizing the expression profiles of *AGAP2-AS1*, LINC01780, and LINC01537 in the TCGA cohort, ranked by patient risk score. **(C–E)** Kaplan-Meier progression-free survival (PFS) curves based on TCGA clinical data, stratifying patients by high (red) versus low (blue) expression of *AGAP2-AS1* **(C)**, LINC01780 **(D)**, and LINC01537 **(E)**. P-values were determined by the log-rank test. **(F–H)** Probability density distribution plots of gene expression for AGAP2-AS1 **(F)**, LINC01780 **(G)**, and LINC01537 **(H)**. Vertical dashed lines indicate optimal risk stratification cutoffs. **(I,J)** Scatter plots correlating mRNA expression with absolute somatic copy number (derived from TCGA) for *AGAP2-AS1* **(I)** and LINC01780 **(J)**. *AGAP2-AS1* shows a significant positive correlation (P = 0.0011), validating its copy-number-driven expression status. **(K, L)** Scatter plots illustrating the correlation between *AGAP2-AS1* expression and ssGSEA enrichment scores for Oxidative Stress Response **(K)** and Cellular Response to UV **(L)**. The Spearman correlation coefficient (R) and P-value are indicated.

We next sought to discern whether this upregulation was structurally driven by genomic amplification. Integrating matched absolute copy number variation (CNV) data from ASCAT2, we observed a modest but statistically significant positive correlation between *AGAP2-AS1* expression and somatic copy number (R = 0.17, P = 0.0011), suggesting that genomic dosage may partially contribute to its deregulation (Figure 4I). A consistent positive trend was also observed for LINC01780 (Figure 4J).

To test our specific hypothesis regarding the genotoxic microenvironment of eyelid tumors, we *a priori* selected canonical UV response (GO:0009636) and oxidative stress response (GO:0006979) pathways for single-sample Gene Set Enrichment Analysis (ssGSEA). *AGAP2-AS1* expression displayed a modest but statistically significant positive correlation with the cellular response to UV radiation (R = 0.19, *P* < 0.001) and a negative correlation with the oxidative stress response pathway (R = −0.26, P < 0.001) (Figures 4K,L). These suggestive associations imply that *AGAP2-AS1* modulation is intricately tied to environmental stress adaptation.

### 2.5 siRNA-mediated knockdown of *AGAP2-AS1* impairs tumorigenic potential *in vitro*

To substantiate the oncogenic functionality of *AGAP2-AS1* inferred from multi-omic screening, we validated its expression pattern and biological necessity *in vitro*. *AGAP2-AS1* abundance was significantly elevated in tumor cells compared to normal controls (*P* < 0.0001; Figure 5A). Transcriptional depletion via small interfering RNAs (siRNAs) yielded robust knockdown efficiency in both A375 (*P* < 0.0001; Figure 5B) and A431 (*P* < 0.0001; Figure 5C) melanoma lines. Phenotypic assessment indicated that *AGAP2-AS1* promotes malignant growth and *in vitro* motility. CCK-8 proliferation assays revealed that *AGAP2-AS1* depletion significantly suppressed the growth trajectories of both A375 and A431 cells, with significant growth inhibition manifesting at 72 and 96 h post-transfection (Figures 5D,E). Crucially, silencing *AGAP2-AS1* compromised cell motility: wound healing assays confirmed a quantifiable delay in scratch closure rates in knockdown groups compared to controls (A375: *P* = 0.0042; A431: *P* = 0.0010; Figures 5F,G). While wound healing assays are intrinsically influenced by concurrent changes in cell proliferation, Transwell assays—which more specifically model matrix penetration—demonstrated a substantial reduction in the infiltrative capacity of tumor cells upon *AGAP2-AS1* knockdown (A375: *P* = 0.0038; A431: *P* = 0.0002; Figures 5H,I). Collectively, these data provide experimental evidence that *AGAP2-AS1* functions as a key mediator of proliferation, migration, and invasion.

**FIGURE 5:**
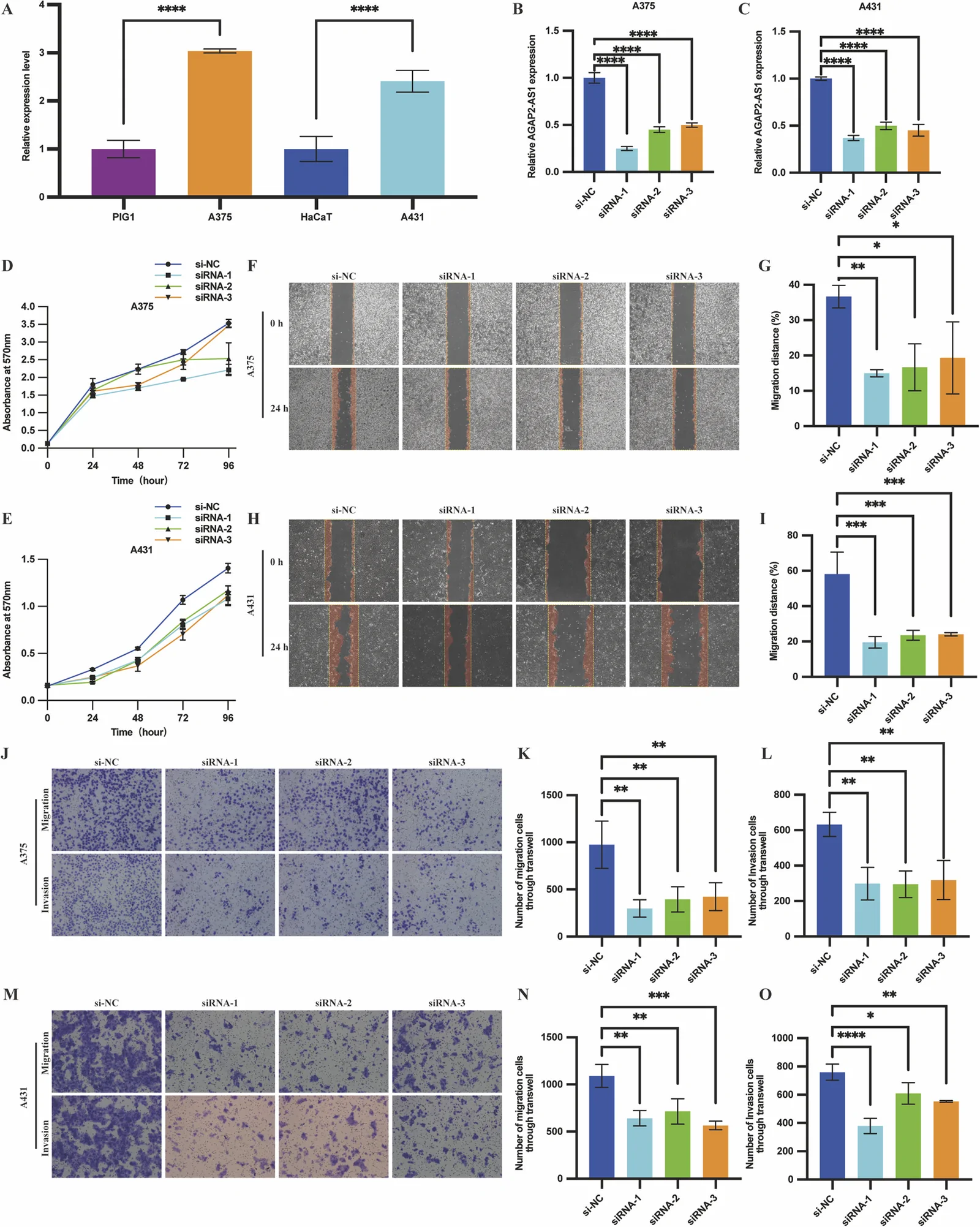
Experimental validation of *AGAP2-AS1* as a functional oncogene driving proliferation, migration, and invasion. **(A)** RT-qPCR analysis quantifying relative *AGAP2-AS1* expression in normal versus tumor cells (*P* < 0.0001). **(B,C)** Validation of knockdown efficiency in A375 **(B)** and A431 **(C)** cell lines transfected with negative control (si-NC) or *AGAP2-AS1*-specific siRNAs. Transcript levels were normalized to GAPDH (*P* < 0.0001). **(D,E)** CCK-8 proliferation assays assessing the viability of A375 **(D)** and A431 **(E)** cells following *AGAP2-AS1* depletion. Data show a significant reduction in optical density (OD) values in knockdown groups at 72 h and 96 h (**P < 0.01, ***P < 0.001 vs. si-NC group). **(F,G)** Quantitative analysis of wound healing assays in A375 **(F)** and A431 **(G)** cells. The migration index was significantly impaired in the knockdown groups. **(H,I)** Quantification of Transwell invasion assays for A375 **(H)** and A431 **(I)** cells. Silencing *AGAP2-AS1* significantly abrogated the invasive potential of tumor cells. Data are presented as mean ± s.d. Statistical significance was determined by one-way ANOVA (for **(A–C)** and **(F–I)**) or two-way ANOVA (for **(D,E)**), both followed by Dunnett’s multiple comparisons test.

## 3 Materials and methods

### 3.1 Patient specimens and ethical statement

The study protocol was approved by the Ethics Committee of the Tianjin Medical University Eye Hospital (Approval No. 2024KY-55) and conducted in strict adherence to the ethical standards of the Declaration of Helsinki. Written informed consent was obtained from all participants. From August 2024 to July 2025, three patients undergoing curative surgical resection for eyelid malignancies were enrolled. To define the somatic genomic landscape, paired tissue specimens were harvested intraoperatively: the tumor group comprised core neoplastic tissue, while the control group consisted of matched adjacent non-neoplastic skin (excised from the surgical margin, >0.5 cm from the macroscopic tumor boundary). Histopathological evaluation confirmed that the adjacent control tissues were devoid of neoplastic infiltration and significant inflammatory changes. These paired samples (n = 3 pairs) were subsequently processed for Circle-Seq, as well as histological (H&E and Masson trichrome) and immunohistochemical (IHC) analyses.

### 3.2 eccDNA purification and circle-seq library preparation

Extrachromosomal circular DNA (eccDNA) purification was optimized based on the Circle-Seq protocol. Genomic DNA was extracted using the HiPure Tissue DNA Midi Kit (Cat# D3122-03; Magen Biotechnology, Guangzhou, China). Total DNA was subjected to alkaline lysis to denature linear chromosomal DNA and separate circular species from linear DNA, lipids, and proteins. The eccDNA-enriched fraction was purified via ion-exchange column chromatography (Plasmid Mini AX; Cat# 010-50; A&A Biotechnology, Poland). Residual linear DNA was depleted by digestion with Plasmid-Safe ATP-dependent DNase (Cat# E3110K; Epicentre, USA), supplemented with the rare-cutting endonuclease MssI (Cat# ER1341; Thermo Scientific, USA) to digest mitochondrial circular DNA (mtDNA) and create accessible ends for exonuclease activity. The eccDNA-enriched templates were amplified via Rolling Circle Amplification (RCA) using phi29 polymerase (REPLI-g Midi Kit; Cat# 150023; Qiagen, Germany). Amplified products were purified using Hieff NGS® DNA Selection Beads (Cat# 12601ES56; Yeasen Biotechnology, China) and sheared by sonication (Bioruptor, Belgium) to an average fragment size of 200–300 bp. Sequencing libraries were constructed using the Hieff NGS® Ultima Pro DNA Library Prep Kit for Illumina® (Cat# 12201ES96; Yeasen Biotechnology) and sequenced on an Illumina® NovaSeq 6,000 platform (PE150 strategy).

### 3.3 Computational identification and annotation of eccDNAs

Raw reads were quality-filtered using Trimmomatic with default parameters. High-quality clean reads (Q30 > 99.9%) were aligned to the human reference genome (hg38; GENCODE v38) using BWA-MEM. High-confidence eccDNAs were identified based on the detection of discordant read pairs and split reads spanning the circularization junction. Candidate circles were annotated against the GENCODE (v38) database to define their genomic context (promoters, exons, introns, and distal intergenic regions). GC content and fragment length distributions were computed using custom R scripts and the Biostrings package.

### 3.4 Integrative multi-omic screening and survival analysis

To prioritize functional drivers from the circle-derived transcriptome, we devised an integrative multi-omic screening framework. We first intersected the in-house eccDNA repertoire with the eccDNA Gene Analyzer (ECGA) database (https://www.zhounan.org/ecga/) to filter for recurrently circularized loci, thereby distinguishing robust biological signals from stochastic noise. The clinical relevance of this filtered repertoire was subsequently interrogated using the Cancer Genome Atlas (TCGA) Skin Cutaneous Melanoma (SKCM) cohort. Transcriptomic profiles (HTSeq-FPKM) and matched clinical data were retrieved via the TCGAbiolinks R package. Prognostic evaluation was performed using univariate Cox proportional hazards regression to calculate Hazard Ratios (HR) and identify candidates significantly associated with progression-free survival (PFS). For the risk-stratified heatmap, patient risk scores were calculated as the linear combination of the expression levels of the selected lncRNAs multiplied by their respective Cox regression coefficients (β = ln (HR)). For Kaplan-Meier survival analysis, patients were stratified into high and low expression groups using maximally selected rank statistics (via the surv_cutpoint function in the survminer R package) to explore maximal prognostic separation. We acknowledge that this optimal cutoff approach is exploratory and inherently inflates statistical significance compared to pre-defined median thresholds. Finally, to substantiate a copy-number-driven expression model, absolute somatic copy number data (ASCAT2) were integrated with gene expression profiles, and linear regression analyses were conducted to verify that upregulation was dictated by genomic dosage.

### 3.5 Functional enrichment analysis

Gene Ontology (GO) and KEGG pathway enrichment analyses for eccDNA-harbored genes were conducted using clusterProfiler with a Benjamini–Hochberg adjusted P-value cutoff of <0.05. Gene Set Enrichment Analysis (ssGSEA) was employed to infer pathway activity scores using the GSVA package. Rather than performing an unbiased global screen, we *a priori* selected canonical UV radiation response (GO:0009636) and oxidative stress response (GO:0006979) pathways to specifically test the hypothesis that *AGAP2-AS1* modulation is linked to the unique genotoxic microenvironment of eyelid tumors. These pathway activity scores were correlated with *AGAP2-AS1* expression using Spearman’s rank correlation.

### 3.6 Cell culture and transfection

Human melanoma cell line A375 and epidermoid carcinoma cell line A431 were cultured in Dulbecco’s Modified Eagle’s Medium (DMEM; Cat# C11995500BT; Gibco, China) supplemented with 10% Fetal Bovine Serum (FBS; Cat# FND500; ExCell, China) and 1% penicillin/streptomycin (Cat# 15140122; Gibco, USA) at 37 °C in a humidified 5% CO2 atmosphere. For loss-of-function assays, cells were transfected with specific small interfering RNAs (siRNAs) targeting *AGAP2-AS1* or a non-targeting negative control (si-NC) synthesized by OBiO Technology (Shanghai, China). Transfections were performed using Lipofectamine 3,000 (Invitrogen) following the manufacturer’s protocol. The specific siRNA target sequences are listed in Supplementary Table S2.

### 3.7 RNA isolation and quantitative real-time PCR (RT-qPCR)

Total RNA was extracted using the EZ-press RNA Purification Kit (Cat# B0004DP; EZBioscience, United States). Reverse transcription was performed using the Color Reverse Transcription Kit (Cat# A0010CGQ; EZBioscience) at 42 °C for 15 min and 95 °C for 30 s. RT-qPCR was conducted on a LightCycler® 480 II instrument (Roche, Switzerland) using the 2× Color SYBR Green qPCR Master Mix (Cat# A0012-R2; EZBioscience). Thermocycling conditions included an initial denaturation at 95 °C for 5 min, followed by 40 cycles of 95 °C for 10 s and 60 °C for 30 s. Relative expression levels were calculated using the 2^^(−ΔΔCt)^ method, normalized to GAPDH. Primer sequences are listed in Supplementary Table S1.

### 3.8 Cell proliferation and motility assays

Cell viability was assessed using the Cell Counting Kit-8 (CCK-8; Dojindo) at 0, 24, 48, 72, and 96 h post-transfection. Cell migration and invasion capabilities were assessed using Transwell® permeable supports (6.5 mm insert, 8.0 µm pore size, Polycarbonate membrane; Cat# 3422; Corning, United States). For the invasion assay, the upper chambers were pre-coated with Matrigel (BD Biosciences) to form a matrix barrier, whereas for the migration assay, the chambers remained uncoated. In both assays, cells (5 × 10^4) suspended in 200 µL of serum-free medium were seeded into the upper chamber, and 600 µL of medium containing 20% FBS was added to the lower chamber to induce chemotaxis. After incubation for 24 h, cells remaining on the upper surface were removed. Cells that had migrated or invaded through the membrane were fixed with 4% paraformaldehyde, stained with 0.1% crystal violet, and quantified in five randomly selected fields using an inverted microscope.

### 3.9 Statistical analysis

All statistical analyses were performed using R software (version 4.2.0) and GraphPad Prism (version 9.0). Continuous variables are presented as mean ± standard deviation (s.d.). For experiments comparing multiple independent treatment groups to a single control group (e.g., evaluating three distinct siRNAs versus a negative control), a one-way analysis of variance (ANOVA) followed by Dunnett’s multiple comparisons test was strictly applied to prevent the inflation of Type I error rates. For time-course proliferation assays (CCK-8), a two-way ANOVA followed by Dunnett’s multiple comparisons test was utilized to evaluate differences between the treatment groups and the control at specific time points. A P-value <0.05 was considered statistically significant.

## 4 Discussion

The topological plasticity of the cancer genome extends far beyond linear chromosomal alterations. While extrachromosomal circular DNA (eccDNA) has historically been viewed as a stochastic byproduct of genomic instability, our findings support its role as a selective, adaptive reservoir of functional non-coding elements in periocular malignancies. By integrating Circle-Seq profiling of diverse eyelid tumors with large-scale genomic cohorts, our data suggest that eccDNA biogenesis is not merely a random shattering event but a process that preferentially amplifies GC-rich, transcriptionally active chromatin domains. Critically, we identify *AGAP2-AS1* as an eccDNA-associated oncogene, supporting a model where focal amplification of this lncRNA confers a survival advantage under environmental genotoxic stress.

Our observation of a pervasive, GC-rich eccDNA landscape in tumor tissues aligns with the “accessible chromatin model” proposed by Wu et al. (2019). In the context of eyelid tumorigenesis, where UV radiation induces constant double-strand breaks, we posit that open chromatin regions—typically rich in gene promoters and regulatory elements—are structurally predisposed to excision and circularization during repair failures. This contrasts with the random fragmentation observed in apoptotic cells, suggesting that the tumor-specific eccDNA repertoire reflects a history of “scarring” from adaptive DNA repair (Møller et al., 2018). The recurrence of hotspots on chromosomes 9, 16, and 20 across distinct histological subtypes (BCC, IFK, MCC) further implies that specific genomic loci possess intrinsic structural fragility or are under strong positive selection during the early stages of periocular neoplastic evolution (Glover et al., 2017).

A notable finding of this work is the identification of AGAP2-AS1 as a copy-number-dependent driver. While AGAP2-AS1 dysregulation has been reported in glioblastoma and NSCLC, previous studies have largely attributed its upregulation to epigenetic modifications or transcriptional trans-activation (Li et al., 2016; Schmitt and Chang, 2016). Our multi-tiered screening strategy reveals a distinct structural mechanism: somatic circularization and subsequent focal amplification. The statistically significant positive correlation between AGAP2-AS1 expression and somatic copy number in the TCGA cohort (Figure 4I) supports a “gene dosage” model, where eccDNA acts as a mobile amplifier to bypass the transcriptional limits of the linear genome (Turner et al., 2017). Although this correlation is modest in magnitude (R = 0.17), likely reflecting the cellular heterogeneity of bulk tumor tissue wherein stromal admixture dilutes tumor-cell-specific expression signals, orthogonal validation in an independent panel of 47 skin cancer cell lines from the Cancer Cell Line Encyclopedia (CCLE) yielded a substantially stronger correlation (R = 0.43, P = 0.0023; Supplementary Figure S1A). Notably, the relationship between AGAP2-AS1 expression and copy number exhibits a nonlinear threshold effect: expression remains relatively stable at copy numbers 1–3 and increases appreciably only at copy number ≥4 (Supplementary Figure S1B), suggesting that the linear Pearson correlation underestimates the true dose–response relationship in the majority of tumors that harbor low-level copy number alterations. These convergent findings across independent datasets strongly support a copy-number-driven expression model for AGAP2-AS1, while acknowledging that additional regulatory inputs—including transcriptional and epigenetic mechanisms—likely modulate its overall expression dynamics. This amplification is particularly advantageous in the unique microenvironment of eyelid tumors. As our functional data indicate, AGAP2-AS1 modulation is intricately linked to oxidative stress and UV response pathways (Alexandrov et al., 2013). Interestingly, our ssGSEA analysis revealed a seemingly paradoxical phenomenon: while AGAP2-AS1 expression correlates positively with the cellular response to UV radiation, it exhibits a modest negative correlation with the canonical oxidative stress response pathway. Rather than acting as a classical antioxidant protector, we hypothesize that AGAP2-AS1 amplification facilitates a “double-hit” oncogenic mechanism in the UV-damaged microenvironment. Canonical oxidative stress response programs normally trigger ROS-induced apoptosis or clearance of irreparably damaged cells. High levels of eccDNA-derived AGAP2-AS1 may actively suppress these defensive response pathways, thereby allowing epidermal cells carrying severe UV-induced DNA lesions to evade elimination. This suppression of cellular defense mechanisms creates a permissive environment for the accumulation of genomic instability (Russo et al., 2019).

The distinction between “passenger” and “driver” circles remains a challenge in the field. By intersecting our *in vitro* discovery set with the ECGA database and validating in clinical cohorts, we filtered out stochastic noise to pinpoint *AGAP2-AS1*. However, the mechanism by which *AGAP2-AS1* exerts its oncogenic effects likely extends beyond simple RNA-protein interactions. Given the recent discovery that ecDNAs can function as mobile enhancers hijacking distal promoters (Helmsauer et al., 2020), it is plausible that the *AGAP2-AS1*-bearing eccDNAs also act as *cis*- or *trans*-regulatory hubs, reshaping the 3D chromatin architecture. Future studies utilizing ChIA-PET or Hi-C on purified eccDNAs will be essential to map these long-range interactions.

We acknowledge several limitations in this study. First, our discovery cohort was restricted to one sample per pathological subtype (n = 1 each for BCC, IFK, and MCC), which precludes statistical inference at the individual subtype level. However, the paired tumor-normal design and the convergent evidence from orthogonal validation strategies—including cross-referencing with the ECGA database and independent clinical validation in the TCGA-SKCM cohort—mitigate the risk that the reported eccDNA signals represent stochastic false positives. Consequently, while the current design successfully captures pan-histological eccDNA features, it cannot resolve subtype-specific circular signatures. Second, our functional validation relied on linear knockdown strategies. Although effective, this approach does not specifically deplete the circular form of AGAP2-AS1. Developing CRISPR-based systems targeting circle-specific junction sequences would provide definitive proof of circle-dependency (Pourrajab and Zare-Khormizi, 2022; Hung et al., 2021). Finally, while we establish the *in vitro* dependency of tumor cells on AGAP2-AS1, *in vivo* models are required to assess the stability and segregation patterns of these eccDNAs during metastasis.

In summary, this study constructs a high-resolution map of the eccDNA landscape in periocular malignancies and highlights *AGAP2-AS1* as a key beneficiary of extrachromosomal amplification. By elucidating the link between circular DNA topology, lncRNA dosage, and environmental stress adaptation, our findings suggest that targeting the non-coding eccDNA transcriptome may represent a novel therapeutic avenue for preventing malignant progression in UV-associated skin cancers.

## Data Availability

The raw sequencing data and processed data generated in this study have been deposited in the National Genomics Data Center (NGDC), China National Center for Bioinformation, Genome Sequence Archive for Human (GSA-Human), under BioProject accession PRJCA074279 (GSA-Human accession: subHRA033073). The data are available under controlled access; access requests can be made through the GSA-Human Data Access Committee (DAC: HDAC010392). All other data supporting the findings of this study are available within the article and its Supplementary Materials.
