# Supplementary material for: Extrachromosomal circular DNA drives focal amplification of AGAP2-AS1 to promote cutaneous tumorigenesis
Source: Front Pharmacol. 2026 Oct 14;17:1906797. doi: 10.3389/fphar.2026.1906797 (PMC13713694; doi:10.3389/fphar.2026.1906797)
Supplement: Supplementary file 1 [file DataSheet1.pdf]

Supplementary Material

1 Supplementary Figures and Tables

Table S1. RT-qPCR primers.

Primer	Sequence
AGAP2-AS1-F	5'-TACCTTGACCTTGCTGCTCTC-3'
AGAP2-AS1-R	5'-TGTCCCTTAATGACCCCATCC-3'
GAPDH-F	5'-GATGCTGGCGCTGAGTACG-3'
GAPDH-R	5'-GCTAAGCAGTTGGTGGTGC-3'

Table S2. siRNA sequences.

siRNA name	siRNA sequence (5'-3')
sh-AGAP2-AS1-1	GACACGAUCAGGAACUCAATT
sh-AGAP2-AS1-2	GCGGGUAGACCCAGAGCAATT
sh-AGAP2-AS1-3	CUUGUUACCUGCUUUAUAATT
sh-NC	UUCUCCGAACGUGUCACGUTT

Figure S1.

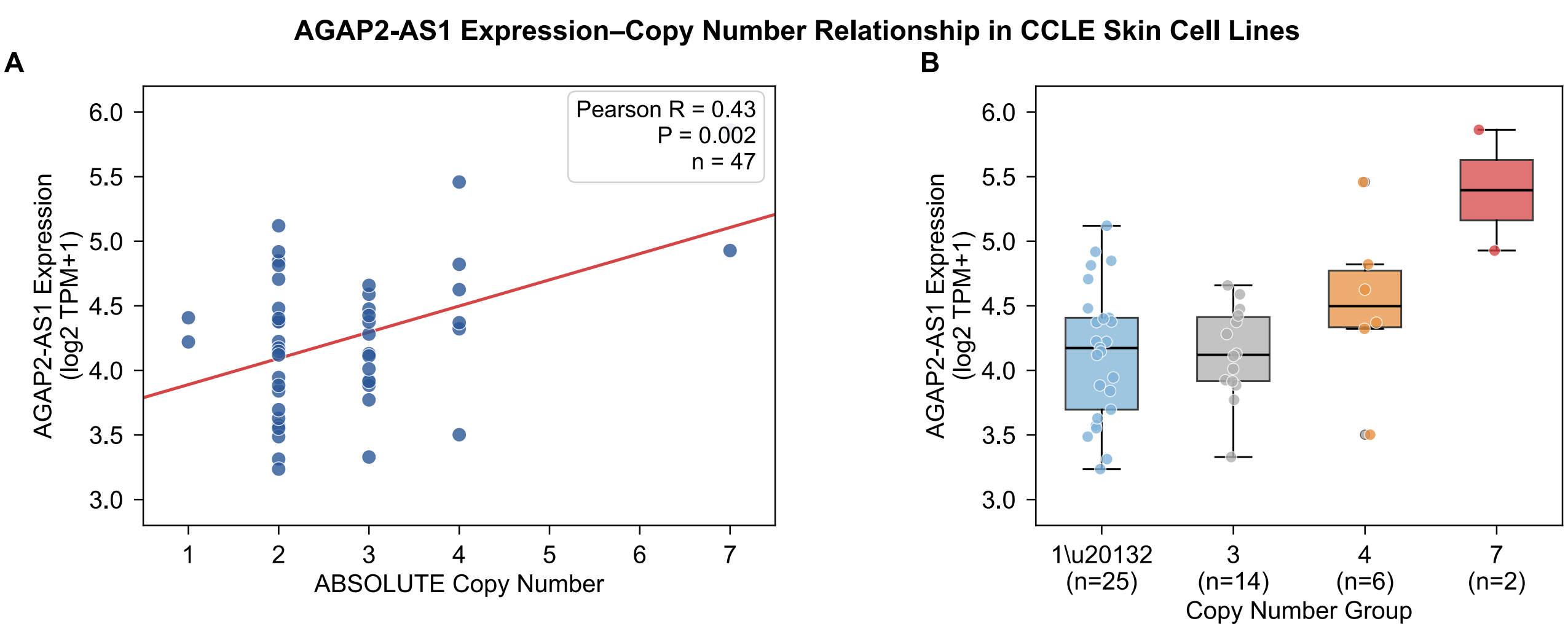

Figure S1. Independent cell line validation of the AGAP2-AS1 expression–copy number relationship. (A) Scatter plot of AGAP2-AS1 expression (log2 TPM + 1) versus ABSOLUTE copy number at the 12q14.1 locus in 47 skin cancer cell lines from the Cancer Cell Line Encyclopedia (CCLE). The

Pearson correlation coefficient (R = 0.43, P = 0.0023; n = 47) and regression line (red) are shown. (B) Box plot of AGAP2-AS1 expression stratified by copy number group. Expression remains comparable across CN 1–4 and increases markedly at CN 7, consistent with a nonlinear dose–response threshold. Individual data points are overlaid as gray dots. The Kruskal–Wallis test P value is indicated above the plot.
